# Can nudge interventions targeting healthy food purchases in real-world grocery stores reduce diet-related health disparities? A pooled analysis of four controlled trials

**DOI:** 10.1186/s12966-024-01687-3

**Published:** 2024-12-03

**Authors:** Josine M. Stuber, Joline WJ. Beulens, Guadalupe X. Ayala, Sarah R. Crozier, S. Coosje Dijkstra, Shih-Fan Lin, Christina Vogel, Joreintje D. Mackenbach

**Affiliations:** 1 Amsterdam UMC location Vrije Universiteit Amsterdam, Epidemiology and Data Science, De Boelelaan 1117, Amsterdam, Netherlands; 2 Amsterdam Public Health, Amsterdam, The Netherlands; 3 Upstream Team, Amsterdam UMC, De Boelelaan 1117, Amsterdam, the Netherlands; 4 School of Public Health, Division of Health Promotion and Behavioral Sciences, San Diego State University, San Diego, United States of America; 5 Institute for Behavioral and Community Health, San Diego State University Research Foundation, San Diego, United States of America; 6 Medical Research Council Lifecourse Epidemiology Centre, University of Southampton, Southampton General Hospital, Tremona Road, Southampton, UK; 7 NIHR Applied Research Collaboration Wessex, Southampton Science Park, Innovation Centre, 2 Venture Road, Chilworth, Southampton, SO16 7NP UK; 8 NIHR Southampton Biomedical Research Centre, University of Southampton and University Hospital Southampton NHS Foundation Trust, Southampton General Hospital, Tremona Road, Southampton, SO16 6YD UK; 9 Public and Occupational Health, Amsterdam UMC location University of Amsterdam, Meibergdreef 9, Amsterdam, Netherlands; 10 Centre for Food Policy, City, University of London, Myddelton Street, London, UK; 11 National Institute for Health and Care Research Southampton Biomedical Research Centre, University of Southampton and University Hospital Southampton NHS Foundation Trust, Tremona Road, Southampton, UK

**Keywords:** Choice architecture, Supermarket, Socioeconomic status, Socioeconomic position, Public health, Prevention

## Abstract

**Background:**

Healthy food nudges may be more, or especially, effective among groups experiencing socioeconomic disadvantage. We investigated the modifying role of socioeconomic and demographic characteristics in the effectiveness of nudge interventions targeting healthy foods in real-world grocery store settings on food purchasing patterns.

**Methods:**

We pooled individual participant data from multiple trials. Eligible trials were identified via a PubMed search and selected based on having a controlled real-world design, testing a nudging intervention promoting healthy purchases, while collecting participants’ sociodemographic and purchasing data. Out of four eligible trials, three had longitudinal measurements, one consisted of a single time point, two were randomised and two were not. Applied nudges consisted of a combination of placement nudges (focussing on availability or positioning) and property nudges (presentation and/or information). Harmonised data included dichotomised socioeconomic and demographic variables and the percentage of purchased fruits and vegetables of total purchases. Multilevel meta-regression based on linear mixed-effects models were used to explore modifying effects using two approaches: longitudinal and cross-sectional analyses.

**Results:**

The analytical sample in the longitudinal analysis comprised of 638 participants, who were predominantly female (76.3%), had a lower education attainment (67.7%), and a mean age of 46.6 years (SD 13.5). These characteristics were similar in the cross-sectional analysis (*n* = 855). Compared to control group participants, there was no main effect of healthy food nudges on the percentage of fruit and vegetable purchases by intervention group participants in the longitudinal analysis (β = 0.00; 95%CI -0.03, 0.09). This main effect was not modified by educational attainment (β_group*higher education_ = -0.06; -0.40, 0.02), sex (β_group*females_ = 0.13; -0.00, 0.61) nor age (β_group*older adults_ = -0.05; -0.39, 0.02). Results from the cross-sectional analysis were comparable.

**Conclusions:**

This pooled analyses of four controlled trials did not find evidence supporting the hypothesis that grocery store nudge interventions of healthy foods work more effectively among groups experiencing socioeconomic disadvantage. Future studies are needed to address the identified limitations through rigorous trial design using comprehensive interventional strategies, standardised outcome measures, while also evaluating context-specific approaches. Such insights will help to better understand the equity of nudging interventions in grocery store settings and the potential for reducing diet-related health disparities.

**Trial registrations:**

The trial of Ayala et al. (2022) was retrospectively registered at ClinicalTrials.gov (NCT01475526; at 14 November 2011, https://clinicaltrials.gov/study/NCT01475526), the of Huitink et al. (2020) was retrospectively registered in the ISRCTN registry (ISRCTN39440735; at 5 September 2018, 10.1186/ISRCTN39440735), the of Vogel et al. (2024) was retrospectively registered at ClinicalTrials.gov (NCT03518151; at 24 April 2018, https://clinicaltrials.gov/study/NCT03518151), and finally of Stuber et al. (2024) was registered in the Dutch Trial Register (ID NL7064, at 30 May 2018, https://www.onderzoekmetmensen.nl/en/trial/20990).

**Supplementary Information:**

The online version contains supplementary material available at 10.1186/s12966-024-01687-3.

## Background

Poor diet quality is linked to obesity and increased chronic disease risk [1, 2]. Additionally, there are continuing diet-related health disparities, where disadvantaged groups—often characterised as having lower educational attainment, occupational position, and/or income [3]—more often have poorer diet quality and a higher burden of disease [4–6]. These poorer diets are driven by a range of factors including the environment in which choices are made [7, 8]. An important food choice environment is the grocery store. Although grocery stores are a prominent source of healthy products, they often promote and stock a higher proportion of unhealthy products, which further undermines the diet quality of individuals, especially those from disadvantaged backgrounds [9]. Given that most foods and beverages are obtained from grocery stores, improving the availability and promotion of healthy options in these environments is crucial to addressing dietary inequities and improving population-wide diet quality [10].

Nudge interventions, small environmental changes to make certain choices more likely without eliminating alternative choices, are one type of alteration to the grocery store which can be aimed at enhancing the selection of healthy choices [11]. Examples include replacing unhealthy snacks at check-out counters with healthy snacks, or placing healthier products in prominent locations such as store entrances or aisle-ends. Results from experimental laboratory studies suggest that nudges are a promising intervention to promote the selection of healthier product choices, although real-world grocery store trials demonstrate that effect sizes are generally small (about 1–2% change in purchasing behaviours) [12–15].

Grocery store nudges target heuristic choices (i.e., simple mental procedures facilitating fast decision making) and instinctive, automatic, purchasing decisions [16]. Pre-existing motivation to change purchasing decisions is thus not required to potentially benefit from the nudges. As such, nudges have a wide reach across populations, including individuals experiencing socioeconomic disadvantage [17–19]. These individuals often face more scarcity of time and resources (e.g., money, social support) [20, 21], which can increase reliance on the instinctive and automatic purchasing decisions [22, 23]. It can therefore be hypothesised that nudges may be more, or especially, effective among groups experiencing socioeconomic disadvantage [24].

Although there is observational evidence that socially disadvantaged groups are for instance more likely to be exposed to a higher number of unhealthy food products in grocery stores [25], the evidence for potential differential effectiveness of nudges across socioeconomic groups is limited and mixed [15, 18, 26–29]. For example, a meta-analysis focussing on both controlled and real-world settings observed that increasing healthy food availability resulted in an equally positive effect across socioeconomic groups [27]. In contrast, a systematic review focussed solely on real-world purchasing settings showed that nudges may be more effective for individuals experiencing socioeconomic disadvantage, but that evidence was of moderate to weak quality [15]. Another systematic review reported that placement nudges led to larger effect sizes among those in disadvantaged groups in real-world purchasing settings [26]. Yet, there is also experimental evidence from a real-world healthy food nudging study suggesting that nudges could indeed benefit individuals living in more disadvantaged areas, but could simultaneously negatively impact those living in more advantaged areas [29].

Moreover, there is little evidence on potential differential effectiveness of nudges by sex and age groups. For example, women are generally more often responsible for the household groceries, leading to increased intervention exposure. Yet, they may also be more health-conscious in their grocery shopping decisions than males [30]. However, whether this leads to reduced or increased effectiveness of nudging healthy products among females is currently unclear. There is some evidence suggesting larger effects in males [31], while this effect is not reported in other grocery store studies [32, 33]. In addition, there is evidence from real-world grocery store settings suggesting increased susceptibility to nudges in older adults [32, 34]. This might be explained by increasing susceptibly to external cues among older individuals [35], but further evidence is lacking.

More high-quality evidence from real-world settings is thus needed to evaluate whether nudging interventions in grocery stores may have differential effects according to various socioeconomic and demographic characteristics of store customers (i.e., participants). Yet, trials evaluating nudge effectiveness in real-world grocery store settings generally rely on small sample sizes, rendering insufficient power for subgroup analyses [36]. This situation hinders potential detection of modifying effects by participant socioeconomic and demographic characteristics; hence, pooling individual participant data from various trials can offer an advantageous solution [37]. As such, the primary objective of this study is to investigate the modifying role of participants’ educational attainment in the effectiveness of nudge interventions in real-world grocery store settings on food purchasing patterns. Secondary objectives include investigating the modifying role of age and sex in the effectiveness of nudge interventions on food purchasing patterns, and the modifying role of educational attainments, age and sex in the effectiveness of nudge interventions on participants’ diet quality.

## Methods

### Study design

We pooled individual participant data from multiple controlled trials (CT) or randomised controlled trials (RCT) investigating the effects of nudging healthy product purchases in real-world grocery stores. A retrospective data harmonisation approach was used [38]. The study was pre-registered in Open Science Framework [39].

### Trial selection

PubMed was searched on March 30, 2023, to identify potential eligible trials, using the search terms *supermarket*, *store*, *retail*, *grocery*, and *nudge*, *choice architecture*, *swap*, *position*, *placement*, *promotion*, and *intake*, *diet*, *sales*, *purchases* (Additional file 1: Supplementary text 1). We selected eligible trials based on the following criteria:

- used a CT or RCT design;
- tested a nudging intervention promoting healthy purchases (where nudging is defined as small changes in the choice environment to make a certain choice more likely without eliminating the alternative choice);
- conducted in a real-world grocery store setting (establishments primarily engaged in retailing foods and beverages [40]);
- collected participant-level food purchasing and/or dietary intake data; and,
- collected data on participant-level educational attainment, age, and sex.

This search resulted in 47 articles, out of which we identified four eligible trials (Table 1) [31, 41–43]. One of the identified trials was based on a protocol paper which was published by the lead author of the current article [43]. We thus had insights into the data of that specific trial, despite the fact that the effect paper of this trial was published at a later time than the literature search was conducted [32]. On May 17, 2024, the PubMed search was repeated yielding 53 articles. However, no new eligible trials were identified.

**Table 1:** Characteristics of eligible trials for the pooled analyses

Author	Year	Study design and intervention period	Study setting	Participants	Nudge interventions^1^	Intervention implementation fidelity	Targeted food groups	Individual-level measurements as potential modifiers	Outcome measurements	Trial results
Ayala et al. [31, 56]	2022	Cluster-RCT: Baseline data collected between October 2011 and October 2013, with follow-up periods at 6 and 12 months. *Note: months 1–6 implementation by researchers and months 7–12 intervention maintained by store employees.*	16 (8 control and 8 intervention) smaller sized grocery stores, Latino/Hispanic-focused, located in an urban community; San Diego County, California, USA. *Note: These food stores had fewer product categories than traditional grocery stores but more than convenience stores.*	*N* = 369	Placement nudges, focussing on *availability* and *position* (fruits additionally displayed at check-out counter; augmenting fruit and vegetable displays in the produce section). Property nudges, focussing on *presentation* (various point-of-purchase marketing materials, and a marketing campaign), *information* (product labelling, and key behavioural messages), and *functionality* (vegetables included in prepared dishes, cross merchandising of vegetables with fresh meat offerings). Combined with food tastings and employees’ capacity building to place and promote fruits and vegetables in store.	No variations by store. Employee trainings, marketing campaign, and structural changes (100% fidelity), although took more time and funding. At 6-months, intervention versus control stores had significantly more fruit and vegetable promotions, although no differences were observed in the environment by availability and position of fruits and vegetables (low fidelity; not quantified).	Fruits and vegetables.	Educational attainment, sex, age, employment status, home ownership, poverty status, marital status, and household size.	Between-group and within-individual change: Dietary intake (daily cups of fruits, daily cups of vegetables (8-item fruit and vegetable screener and 12-item fat screener)) and purchase outcomes: Weekly $ spent on groceries at targeted store, and on fruits and vegetables at targeted stored. Measured via questionnaires at baseline, at month 6 and at month 12.	Null result: no overall changes in diet and weekly $ spend on fruits and vegetables. Interaction by sex found on fruit consumption over time (i.e., beneficial effects in males, not in females).
Huitink et al. [42]	2020	CT: two Intervention days versus two control days over a two week period in May 2017.	1 grocery store in a socially disadvantaged area, the Netherlands, comparing two intervention versus two control days.	*N* = 244	Property nudge, focussing on *presentation* (inlay shopping trolley) combined with *information* (three variations of a social norm message)	Nudges were implemented on each intervention day by the research team, resulting in an implementation fidelity of 100%.	Vegetables.	Educational attainment, sex, and age.	Between-group difference: Purchases measured via cash receipts of single store visit (items).	Positive effect: intervention groups customers were in a higher tertile for vegetables purchases (OR: 1.66, 95% CI: 1.03–2.69), compared to controls.
Vogel et al. [41]	2021	CT (matched, pilot); 6-month intervention period which took place between April 2016 and March 2017.	6 (3 control and 3 intervention) discount grocery stores in socially disadvantaged areas, England.	*N* = 107 (females-only) with loyalty card data; *N* ≈ 138 with diet quality data	Placement nudges, focussing on *availability* (expanding the produce section) and *position* (relocation of frozen vegetables to more prominent place near produce section). Confectionery were removed from check-out counters and the opposite end-of-aisle. Intervention stores also underwent regular store improvements in terms of presentation which were not part of the nudges, including cleaning, painting, and updated signage.	Over the intervention period and across all stores, 100% of interventions were implemented for the fruit and vegetable components. Stock levels of non-food items at checkouts were lower than planned in two intervention stores during the 3-6-month postintervention period.	Fruits and vegetables, and confectionery.	Educational attainment, age, white ethnicity, marital status, employment status.	Between-group and within-individual change: Overall diet quality (20-item FFQ), and daily fruit and vegetable intake (2-item tool: quantity of consumed fruits and vegetables). Purchases via loyalty cards, aggregated to items purchased per week.	Positive effect: proportion weekly fruits and vegetables purchases rose among intervention participants at 3 and 6 months compared to control participants (0.2% versus − 3.0%, *P* = 0.22; 1.7% versus − 3.5%, *P* = 0.05, respectively). But no differences were observed for confectionery purchasing.
Stuber et al. [32, 43]	2024	Cluster-RCT; 6 to 12-month intervention period which took place between January 2021 and May 2022.	12 (6 control and 6 intervention) grocery stores in socially disadvantaged areas, the Netherlands.	*N* = 217 with loyalty card data; *N* = 361 with diet quality data	Placement nudges, focussing on *availability* and *position* (fruits and vegetables additionally displayed at check-out counter). Property nudges, focussing on *presentation* (various point-of-purchase marketing materials) and *information* (shelf-labels, which highlighted the product’s *tastiness*, *convenience*, or *popularity*). Combined with a few pricing strategies.	Over the intervention period and across all stores, 72% of interventions were implemented by the store employees as planned.	Various food groups, including fruits and vegetables.	Educational attainment, sex, and age.	Between-group and within-individual change: Overall diet quality (40-item FFQ), and weekly food and beverage purchases via loyalty cards (grams).	Null result: no changes in both diet quality and weekly purchases. Interaction by age found on diet quality over time (i.e., larger effects in older age).

^1^Nudges classified according to the “typology of interventions in proximal physical micro-environments” framework by Hollands et al. (2017)

CT = controlled trial, RCT = randomised controlled trial

### Eligible trial characteristics

All eligible trials targeted socially disadvantaged populations and collected data on educational attainment as a proxy for experiencing socioeconomic disadvantage (Table 1). One study included a female only sample [41]. One trial was conducted during the COVID-19 pandemic [32], while the others ran prior to this period [31, 41, 42]. All trials studied the effect of certain combination of nudges, and we classified the applied nudges according to the “Typology of interventions in proximal physical micro-environments” framework [44]. Most applied a combination of placement nudges (availability and position nudges) and property nudges (mostly presentation nudges and some information nudges) [31, 32, 41], although one trial studied a property nudge (which consisted of a presentation nudge combined with a social norm nudge) [42] and another specifically used a property nudge which consisted of changing how fruits and/or vegetables were offered (raw, ready-to-cook, ready-to-eat) [31]. Three trials focussed on fruits and/or vegetables as a study outcome [31, 41, 42], and one focussed on multiple food groups [32]. Operationalisation of study outcomes regarding fruit and vegetable purchases were based on weekly dollars spent [31], or on grams or items purchased [32, 41, 42]. Three trials were based on a longitudinal design, measuring within-subject and between-subject changes over time [31, 32, 41]. The fourth trial was based on a between group comparison, at a single time point [42].

### Data collection

De-identified individual participant data were collected from the corresponding authors of each individual trial. Upon this data request, these authors were also invited to join the research team for the current article. Prior to actual data transfer, the receiving institute of the lead author signed data transfer agreements which were provided by each of the three data transferring institutes. Received data were centrally stored at protected servers of the receiving institute. Collected datasets contained the following variables: study participant code; grocery store code (if applicable); group allocation; time variable indicating measurement time point (if applicable); socioeconomic and demographic variables; trial-specific confounding variables (see description at ‘data harmonisation’); and items or grams purchased or money spent on fruits and vegetables, total items or grams purchased or money spent per purchase; and diet quality score (if available).

### Data quality assessment

We performed consistency checks of the received datasets by inspecting the data for missing values and running descriptive statistics of participant characteristics. Observations were cross referenced with the published results of each individual trial and no discrepancies emerged.

### Data harmonisation

Data were harmonised according to the recoding scheme presented in Additional file 1: Table S1. The percentage of fruit and vegetable purchases of total purchases was calculated either based on items or grams purchased or on money spent. Diet quality scores were standardised via z-scores following the approach used in one of the trials [41]. The socioeconomic and demographic variables were dichotomised to obtain a sufficient number of participants in each category, where educational attainment was dichotomised as lower (low and medium level educational attainment) and higher (higher educational attainment) (Additional file 1: Table S1). Age was dichotomised into younger adults (18–55 years) and older adults (> 55 years) following the categories used in one of the trials [42]. Relevant confounding variables available across trials were sex, age, education (i.e., for the analysis in which they are not considered a modifying variable), and number of persons purchased groceries for. For two trials, the variable ‘household size’ was used to represent ‘number of persons purchased groceries for’ [32, 41].

Regarding the harmonisation of time points, data were analysed by two different methodologies to take into account the fact that one out of four trials collected all data at one point in time [42], while the others collected longitudinal data (Additional file 1: Table S2) [31, 32, 41]. First, a longitudinal analysis was conducted based on the repeated measurements used in three of the trials [31, 32, 41]. Second, a cross-sectional analysis was conducted including all data from the fourth trial [42], combined with data of the longitudinal trials collected at baseline (as control group data) and follow-up month 6 (as intervention group data) [31, 32, 41].

### Statistical analyses

We report the population characteristics stratified by group to visually inspect potential differences between the intervention and control groups. Normally distributed continuous variables are presented using mean (SD), non-normally distributed continuous variables using median (IQR), and categorical variables using frequencies (percentage).

A multilevel meta-regression approach was applied [37, 45]. The main outcome, percentage of fruit and vegetable purchases, appeared to have a somewhat right-skewed data distribution. The squared root transformation was used to satisfy the normality assumption. Results thus reflect back-transformed means from the square root transformations.

The longitudinal analysis was based on a three-level linear mixed-effects model, using the group variable as the independent variable and the percentage of fruit and vegetable purchases, or diet quality, as the dependent variable. We included random intercepts for trials (i.e., the four included studies), grocery stores, and participants, to account for clustering of participants within trials, and within grocery stores, and the repeated measurements within participants, respectively. We tested if adding random slopes for treatment, grocery stores, and participants significantly improved the model fit based on the likelihood ratio test. Random slopes did not improve model fit and were thus not included. Interaction between time and group indicated no time-specific intervention effects so this interaction was left out of the model. The fixed effect part of the model also included the following covariates: measurement timepoint (categorical), the baseline measurements of the outcome (purchases or diet quality), and the relevant confounding variables which were available across trials. The cross-sectional analysis followed the same approach, with the exception of the random intercept for participants and the covariates measurement timepoint and the baseline measurements of fruit and vegetable purchases. Model fits were evaluated with Q-Q plots.

It was not possible to include trial-specific confounders which were not measured by the other trials (i.e., acculturation, poverty, and homeownership [31], and money spent on groceries [41]). Therefore, individual data of these studies were analysed via the same model approach as described above, but without a random intercept for trials, to explore the influence of these additional confounders on the direction of the overall intervention effect on the purchasing outcome. No substantial impact on the direction of the effect appeared.

Modifying effects between the nudging interventions and socioeconomic and demographic characteristics on fruit and vegetable purchases or on diet quality, in both the longitudinal and cross-sectional analyses, were assessed via interaction terms between the group variable and educational attainment, age, and sex (all dichotomous). The data from Vogel et al. was limited to females and to younger adults and was thus excluded for the evaluation of modifying effects by sex and the dichotomous variable of age [41]. Since there were only two trials available for analyses with the diet quality outcomes (data by Vogel et al. [41]. and Stuber et al. [32]), modifying effects with the interventions on the diet quality outcome could only be assessed for educational attainment and the continuous variable of age, due to the absence of females and older adults in the data by Vogel et al. In sensitivity analyses, interaction by age on the purchasing outcome was further explored by using the continuous variable of age based on the three trials which had these data available [31, 32, 41].

For completeness, the overall crude and confounding-adjusted pooled effects in the intervention group compared to the control group are presented. The degree of between-study heterogeneity is visually explored via a graphical display of trial-specific results [46]. The primary study results were the beta coefficients and 95% confidence intervals (CI) of the interaction terms. The absence of zero in the 95% CI was deemed a significant interaction. Stratified effects are presented by educational attainment, since this is the primary question of interest. Stratified analyses for sex and age were only conducted in the case of (a trend towards) significant interaction effects.

We did not account for multiple testing since these analyses are of an explorative nature [47]. Participants with missing purchasing data were excluded, as well as those without a baseline purchasing measurement to pair with at least one follow up measurement. Cases with missing data on socioeconomic or demographic variables were also excluded. Analyses were performed in R (version 4.3.2).

## Results

### Study population characteristics

The longitudinal analysis comprised of 917 participants. After excluding those missing purchasing data (*n* = 129), a baseline outcome measurement to pair with at least one follow-up measurement (*n* = 147), and socioeconomic or demographic data (*n* = 3), 638 participants were left in the analytical sample for the fruit and vegetable purchases outcome (Additional file 1: Figure S1). These participants were predominantly female (76.3%), with lower education attainment (67.7%), and a mean age of 46.6 years (SD 13.5). The median percentage of fruit and vegetable purchases of the total purchases at baseline was 25.0% (IQR 20.6). These characteristics were evenly distributed between groups, with the exception for age (dichotomous) (Table 2). Compared to the intervention group, the control group consisted of relatively fewer younger adults.

**Table 2:** Study population characteristics at baseline in the longitudinal data analyses^1^ (*n* = 638)

	Control group (*n* = 332)		Intervention group (*n* = 306)	
Participants within trials, *n* (%)				
*Trial by Ayala et al.*	170	(51.2)	173	(56.5)
*Trial by Vogel et al.*	51	(15.4)	46	(15.0)
*Trial by Stuber et al.*	111	(33.4)	87	(28.4)
Educational attainment,^2^ *n* (%)				
*Lower*	228	(68.7)	204	(66.7)
*Higher*	104	(31.3)	102	(33.3)
Sex, *n* (%)				
*Females*	251	(75.6)	236	(77.1)
*Males*	81	(24.4)	70	(22.9)
Age, *n* (%)				
*Younger adults (18–55 years)*	217	(65.4)	229	(74.8)
*Older adults (> 55 years)*	115	(34.6)	77	(25.2)
Age, mean (SD)	47.6	(13.9)	45.4	(13.1)
Number of persons purchased groceries for, median [IQR]	4.0	[3.0]	4.0	[3.0]
Percentage fruit and vegetable purchases of total purchases, median [IQR]	25.0	[19.3]	25.0	[22.6]
Z-score of diet quality^3^, mean (SD)	-0.0	(1.0)	-0.1	(1.0)

^1^Longitudinal analysis includes data from three of the included trials; ^2^Lower = low and medium level educational attainment, higher = higher educational attainment; ^3^Based on *n* = 496 due to absence of comparable diet quality data in Ayala et al. trial

Regarding the sample for the diet quality outcome (*n* = 917), after excluding those missing diet quality data (*n* = 370), a baseline outcome measurement to pair with at least one follow-up measurement (*n* = 48), and socioeconomic or demographic data (*n* = 3), 496 participants were left for analyses (Additional file 1: Figure S2). The baseline diet quality z-score was on average − 0.1 (SD 1.0) (Table 2).

The cross-sectional analysis consisted of 1,161 participants. After excluding those missing purchasing data (*n* = 274), and socioeconomic or demographic data (*n* = 32), 855 participants were left for analysis (Additional file 1: Figure S3). These participants were predominantly female (72.7%), with a lower educational attainment (60.2%), and a mean age of 47.3 years (SD 13.5). The median percentage of fruit and vegetable purchases relative to total purchases at baseline was 21.4% (IQR 22.4). These characteristics were also evenly distributed between the two groups except for age (dichotomous) (Additional file 1: Table S3).

### Fruit and vegetable purchases in the longitudinal analysis

There was no intervention group main effect of the nudges on fruit and vegetable purchases on average over time (β_adjusted_ = 0.00; 95%CI -0.03, 0.09) (Table 3). Nudge intervention effectiveness was not modified by educational attainment (β_group*higher education_=-0.06; -0.40, 0.02). Also, stratified results did not show differences in effectiveness between participants with lower (β_adjusted_ = 0.00; -0.02, 0.09; *n* = 432) versus higher educational attainment (β_adjusted_=-0.04; -0.28, 0.02; *n* = 206). Effectiveness was not modified by age (β_group*older adults_=-0.05; -0.39, 0.02). A non-significant trend suggested stronger effects among females (β_group*females_ = 0.13; -0.00, 0.61). However, stratified results revealed a small and non-significant negative effect among males (β_adjusted_=-0.07; -0.37, 0.01; *n* = 151) and no effect among females (β_adjusted_ = 0.00; -0.05, 0.08; *n* = 390).

**Table 3:** Nudge effectiveness and modifying effects on fruit and vegetable purchases, in the longitudinal analysis (*n* = 638)^1^

	β	95%CI	*p*-value
Crude intervention group main effect	0.00	-0.04, 0.04	0.94
Adjusted intervention group main effect^2^	0.00	-0.03, 0.09	0.91
Nudge interventions x higher educational attainment^2^	-0.06	-0.40, 0.02	0.20
Nudge interventions x females^2,3^	0.13	-0.00, 0.61	0.09
Nudge interventions x older adults^2,3^	-0.05	-0.39, 0.02	0.25

^1^Main within- and between group nudge effectiveness and modifying effects of group by educational attainment, sex, and age, on the percentage of fruit and vegetables purchased, based on the longitudinal analysis. Analyses were based on a three-level linear mixed-effects model, with group as independent variable and the square root of the percentage of fruit and vegetable purchases as the dependent variable. Further fixed effects were time (categorical) and the baseline measurement of percentage of fruit and vegetable purchases. Random intercepts were included for trials, grocery stores, and participants. Results reflect back-transformed means from the square root transformations

^2^Adjusted for educational attainment, sex, age, and the number of persons purchased groceries for

^3^Based on *n* = 541 due to absence of males and older adults in the data by Vogel et al

### Fruit and vegetable purchases in the cross-sectional analysis

Results from the cross-sectional analysis were similar to those from the longitudinal analysis (Table 4). Educational attainment did not modify the effectiveness of nudges on purchases (β_group*higher education_=-0.00; 95%CI -0.24, 0.16) and the stratified results did not indicate differences in effectiveness between participants with lower (β_adjusted_ = 0.00; -0.08, 0.07; *p*-value 0.94; *n* = 515) versus higher educational attainment (β_adjusted_=-0.00; -0.18, 0.09; *p*-value 0.73; *n* = 340).

**Table 4:** Nudge effectiveness and modifying effects on fruit and vegetable purchases, in the cross-sectional analysis (*n* = 855)^1^

	β	95%CI	*p*-value
Crude difference in the intervention group	-0.00	-0.06, 0.05	0.93
Adjusted difference in the intervention group^2^	-0.00	-0.06, 0.05	0.91
Nudge interventions x higher educational attainment^2^	-0.00	-0.24, 0.16	0.85
Nudge interventions x females^,3^	0.07	-0.05, 0.57	0.29
Nudge interventions x older adults^2,3^	-0.13	-0.68, 0.01	0.12

^1^Main between group differences for nudge effectiveness and modifying effects of group by educational attainment, sex and age, on the percentage of fruit and vegetables purchased, based on the cross-sectional analysis (*n* = 855). Analyses were based on a two-level linear mixed-effects model, with group as the independent variable and the square root of the percentage of fruit and vegetable purchases as the dependent variable. Random intercepts were included for trials and grocery stores. Results reflect back-transformed means from the square root transformations

^2^Adjusted for educational attainment, sex, age, and the number of persons purchased groceries for

^3^Based on *n* = 768 due to absence of males and older adults in the dataset by Vogel et al

### Diet quality in the longitudinal analysis

There was no main effect of the nudge interventions on diet quality on average over time (β_adjusted_ = 0.00; 95%CI -0.13, 0.14) (Table 5). Neither education attainment (β_group*higher education_ = 0.02; -0.20, 0.21), nor age (β_group*age_=-0.00; -0.01, 0.01) modified the effect of the nudge interventions on diet quality.

**Table 5:** Nudge effectiveness and modifying effects on diet quality, in the longitudinal analysis (*n* = 496)^1^

	β	95%CI	*p*-value
Crude intervention group main effect	0.00	-0.13, 0.14	0.97
Adjusted intervention group main effect^2^	-0.02	-0.14, 0.10	0.72
Nudge interventions x higher educational attainment^2^	0.02	-0.20, 0.21	0.87
Nudge interventions x age (continuous)^2^	-0.00	-0.01, 0.01	0.91

^1^Main within- and between group nudge effectiveness and interaction effects of group by the educational attainment and age on diet quality (z-score), based on the longitudinal analysis (*n* = 496). Analyses were based on a three-level linear mixed-effects model, with group as independent variable and diet quality as the dependent variable. Further fixed effects were time (categorical) and the baseline measurement of diet quality. Random intercepts were included for trials, grocery stores, and participants

^2^Adjusted for educational attainment, sex, age, and the number of persons purchased groceries for

### Sensitivity analysis

Exploration of age as a continuous variable to investigate modifying effects of the nudges on fruit and vegetable purchases resulted in similar null results, both in the longitudinal analysis (β_group*age_ = 0.00; 95%CI -0.00, 0.00; *p*-value 0.40; *n* = 638) and cross-sectional analysis (β_group*age_=-0.02; -0.04, 0.00; *p*-value 0.06; *n* = 640). The effectiveness of the nudge interventions did not vary considerably across trials (Additional file 1: Figures S4-S6), since the confidence intervals of trial-specific effects overlapped while effect sizes were very small.

## Discussion

This pooled analysis of four controlled trials showed that socioeconomic and demographic characteristics did not modify the effectiveness of healthy food nudge interventions in real-world grocery stores on fruit and vegetable purchases nor on diet quality. The hypothesis that grocery store nudges work more effectively among groups experiencing socioeconomic disadvantage, and may thereby contribute to reducing diet-related health disparities, is thus not supported by the current findings.

To the best of our knowledge, this was the first pooled analysis of multiple real-world grocery store trials on the effectiveness of nudging healthy product purchases. A key strength was the inclusion of participant-level data resulting in a relatively large sample size to evaluate modifying effects. Additionally, this study used a one-stage multilevel meta-regression to obtain single estimates per outcome while correcting for confounders and clustering of data. External validity was increased by combining data from multiple countries, which were all based on real-world grocery settings. This study, however, was also subject to several limitations. The absence of an overall intervention effect across trials may have attenuated the possibility of detecting modifying effects. Other limitations primarily stem from challenges of data harmonisation due to variabilities across trials. First, applied nudges varied in terms of type and number of nudges implemented, which likely have differential effects across different types of individuals, and study outcomes were measured and operationalised in various ways. In addition, the implementation fidelity varied across trials and the two trials with a 100% implementation fidelity showed positive study results in their published articles [41, 42]. Second, although categorisation of educational attainment followed a similar pattern across trials, the meaning of higher education differed. For example, one trial defined ‘high’ educational attainment as ‘high school degree or more’ given traditional levels of education obtained by the study sample in their home countries [31], but in others a high school degree was considered a ‘medium’ educational attainment [32, 41, 42]. Inconsistency in defining educational attainment across trials may have introduced misclassification, as well as the fact that we could only use a single variable to define socioeconomic disadvantage. Third, the study populations across the four trials differed in number of female versus male participants, where especially males were underrepresented. The results are thus less generalisable to males. Fourth, one trial was conducted among stores with a smaller product mix [31] than in the other three trials [32, 41, 42]. Although these differences led to some heterogeneity, the within-study effects were comparable in terms of effects sizes – suggesting relatively low statistical heterogeneity.

Our findings are in contrast to prior literature mostly suggesting that behaviourally-oriented nudges could favour groups experiencing socioeconomic disadvantage more when compared to groups experiencing advantages [15, 18, 26–28]. The lack of modifying effects for educational attainment may be explained by the lack of a main effect in the pooled outcomes, although an overall null result does not necessarily mean that subgroup effects are also absent. Nevertheless, in the present study, the applied nudges may not have been strong enough to increase healthy purchasing behaviours. The magnitude of the interventions across the included trials were likely not sufficient to counterbalance the extremely high availability, dominant promotion and cheaper pricing of unhealthy products in the grocery stores. Also, the produce section may not have been the most suitable food group to investigate modifying effects, since previous studies have suggested that other food groups such as dairy or wholegrain products may be more susceptible to nudging [29, 48]. Yet, only one of the harmonised trials had evaluated nudge effectiveness across a variety of food groups [32]. Moreover, the nudges aimed to change purchasing decisions within a complex real-world setting. These choices are influenced by numerous factors beyond nudging, such as cultural norms, preferences, psychological and social factors, and economic constraints [49], potentially attenuating intervention effects. Last, the harmonised outcome measures may not have been precise enough to detect subtle intervention effects. Especially since two of the included trials did report a positive intervention effect for their trial-specific outcomes [41, 42]. These differences may highlight genuine differences between countries which have been diminished in the pooled analyses [50].

The present findings have important implications for future research aimed at reducing diet-related health disparities through nudge interventions. First, further evaluation of the equity effects and differential effects of age and sex of healthy food nudging interventions in real-world settings is needed based on trials using a combination of comprehensive nudging intervention strategies, to maximise their impact on purchasing patterns. Intervention strategies should not only focus on the promotion of healthy purchases, but also discourage unhealthy purchases while focussing on promoting substitution behaviours (i.e., substituting unhealthy purchases with healthy purchases). Such interventions can, for example, be achieved via replacing unhealthy product availability at checkout counters with healthy products or non-food products [41], controlling the amount of and/or placement of unhealthy product marketing and promotions, and limiting the availability of unhealthy products within stores [51, 52]. It should be noted that, for sustainable implementation of such strategies, they need to be enforced by governmental policies to create a level playing field for retailers. For instance, in Berkeley, California, USA, a healthy checkout in now a mandatory policy for large food retailers [53] and the UK government is taking initial steps to mandate healthier food marketing policies nationwide, although the legislation progress is moving slowly [54, 55]. Further, evaluations should be based on a wide variety of individuals with differing socioeconomic characteristics, and on high quality and novel trial designs, such as a randomised stepped-wedge approach [36]. The inconsistency in defining educational attainment across trials highlights the importance of using clear and standardised measures of socioeconomic characteristics, such as educational attainment, to accurately assess modifying effects and reduce potential measurement error. In addition, socioeconomic characteristics should ideally be defined based on a combination of variables such as educational attainment, income, and occupation [3]. Ultimately, using such standardised measures will facilitate future studies to pool individual participant data of multiple trials to strengthen the evidence base. Last, the observed heterogeneity due to differences in study settings, interventions applied, and populations targeted underscores the need for future research on the differential effects of nudge interventions across various socioeconomic and regional contexts. For example, larger effects may be expected in settings with a higher presence of groups experiencing socioeconomic disadvantage combined with low availability of healthy food stores and a ban on unhealthy food advertising in public places. Studies designed to detect these effects could contribute to identifying which specific nudging strategies, in which specific contexts, may be most effective in reducing health inequalities.

## Conclusions

While this pooled analyses of four controlled trials did not find evidence supporting the hypothesis that grocery store nudge interventions of healthy foods work more effectively among groups experiencing socioeconomic disadvantage, it provides valuable insights for directing future research and development of intervention strategies. Future studies are needed to address the identified limitations through rigorous trial design using comprehensive intervention strategies, standardised outcome measures, while also evaluating context-specific approaches. Such insights will help to better understand the equity of nudging interventions in grocery store settings and the potential for reducing diet-related health disparities.

## Electronic supplementary material

Below is the link to the electronic supplementary material.

Additional file 1: Supplementary Text 1. Search string used for literature search in PubMed; Table S1. Recoding scheme to generate harmonised dataset; Table S2. Data re-structuring into a longitudinal and a cross-sectional data frame; Table S3. Study population characteristics in the cross-sectional analysis1 (*n* = 855); Figure S1. Flow diagram of exclusions in the longitudinal analysis for the fruit and vegetable purchases outcome; Figure S2. Flow diagram of exclusions in the longitudinal analysis for the diet quality outcome; Figure S3. Flow diagram of exclusions in the cross-sectional data frame for the fruit and vegetable purchases outcome; Figure S4. Trial-specific and overall pooled within- and between group nudge effectiveness on the percentage of fruit and vegetables purchased, based on the longitudinal data frame; Figure S5. Trial-specific and overall pooled between group nudge effectiveness on the percentage of fruit and vegetables purchased, based on the cross-sectional data frame; Figure S6. Trial-specific and overall pooled within- and between group nudge effectiveness on diet quality, based on the longitudinal data frame

## Data Availability

The harmonised datasets will not be made publicly available. Data requests can be sent to the corresponding authors of each individual trial.

## Code Availability

The analytical code supporting the findings of this study is available from the corresponding author upon request.

## Abbreviations

CT: Controlled trial
RCT: Randomised controlled trial

## References

[1] Roth GA, Mensah GA, Johnson CO, Addolorato G, Ammirati E, Baddour LM, et al. Global Burden of Cardiovascular diseases and Risk factors, 1990–2019 Update from the GBD 2019 study. J Am Coll Cardiol. 2020;76(25):2982–3021. 33309175 10.1016/j.jacc.2020.11.010 PMC7755038

[2] Safiri S, Karamzad N, Kaufman JS, Bell AW, Nejadghaderi SA, Sullman MJM et al. Prevalence, deaths and disability-adjusted-life-years (DALYs) due to type 2 diabetes and its attributable risk factors in 204 countries and territories, 1990–2019: results from the global burden of Disease Study 2019. Front Endocrinol. 2022;13. 10.3389/fendo.2022.838027 PMC8915203 35282442

[3] Shavers VL. Measurement of socioeconomic status in health disparities research. J Natl Med Assoc. 2007;99(9):1013–23. 17913111 PMC2575866

[4] Satia JA. Diet-related disparities: understanding the problem and accelerating solutions. J Am Diet Assoc. 2009;109(4):610–5. 19328255 10.1016/j.jada.2008.12.019 PMC2729116

[5] Marmot M, UCL Institute of Health Equity. &. Review of social determinants and the health divide in the WHO European Region: final report: World Health Organization. Regional Office for Europe; Updated reprint 2014 [ https://apps.who.int/iris/handle/10665/108636

[6] WHO. Global status report on noncommunicable diseases 2014 Switzerland: World Health Organization. 2014 [ https://apps.who.int/iris/handle/10665/148114

[7] Glanz K, Sallis JF, Saelens BE, Frank LD. Healthy nutrition environments: concepts and measures. Am J Health Promotion. 2005;19(5):330–3. 10.4278/0890-1171-19.5.330 15895534

[8] Story M, Kaphingst KM, Robinson-O’Brien R, Glanz K. Creating healthy food and eating environments: policy and environmental approaches. Annu Rev Publ Health. 2008;29:253–. 10.1146/annurev.publhealth.29.020907.090926 18031223

[9] Poelman MP, Dijkstra SC, Djojosoeparto SK, de Vet EWML, Seidell JC, Kamphuis CBM. Monitoring van de mate van gezondheid van het aanbod en de promoties Van supermarkten en out-of-home-ketens. Wageningen: Wageningen University & Research; 2021.

[10] Rose G. Sick individuals and sick populations. Int J Epidemiol. 1985;14(1):32–8. 3872850 10.1093/ije/14.1.32

[11] Thaler RH, Sunstein CR, Nudge. Improving decisions about Health, Wealth, and happiness. New Haven: Yale University Press; 2008.

[12] Hartmann-Boyce J, Bianchi F, Piernas C, Riches SP, Frie K, Nourse R, Jebb SA. Grocery store interventions to change food purchasing behaviors: a systematic review of randomized controlled trials. Am J Clin Nutr. 2018;107(6):1004–16. 29868912 10.1093/ajcn/nqy045 PMC5985731

[13] Shaw SC, Ntani G, Baird J, Vogel CA. A systematic review of the influences of food store product placement on dietary-related outcomes. Nutr Rev. 2020. 10.1093/nutrit/nuaa024 PMC7666915 32483615

[14] Vecchio R, Cavallo C. Increasing healthy food choices through nudges: A systematic review. Food Quality and Preference 2019(78).

[15] Harbers MC, Beulens JWJ, Rutters F, de Boer F, Gillebaart M, Sluijs I, van der Schouw YT. The effects of nudges on purchases, food choice, and energy intake or content of purchases in real-life food purchasing environments: a systematic review and evidence synthesis. Nutr J. 2020;19(1):103. 32943071 10.1186/s12937-020-00623-y PMC7500553

[16] Cohen DA, Babey SH. Contextual influences on eating behaviours: heuristic processing and dietary choices. Obes Rev. 2012;13(9):766–79. 22551473 10.1111/j.1467-789X.2012.01001.x PMC3667220

[17] Marteau TM, Hollands GJ, Fletcher PC. Changing human behavior to prevent Disease: the importance of targeting automatic processes. Science. 2012;337(6101):1492–5. 22997327 10.1126/science.1226918

[18] McGill R, Anwar E, Orton L, Bromley H, Lloyd-Williams F, O’Flaherty M et al. Are interventions to promote healthy eating equally effective for all? Systematic review of socioeconomic inequalities in impact. BMC Public Health. 2015;15. 10.1186/s12889-015-1781-7 PMC4423493 25934496

[19] Adams J, Mytton O, White M, Monsivais P. Why are some Population interventions for Diet and obesity more Equitable and Effective Than others? The role of Individual Agency. PLoS Med 2016;13(4). 10.1371/journal.pmed.1001990 PMC4821622 27046234

[20] Mullainathan. Sendhil, Shafir. Scarcity: why having too little means so much. New York: Henry Holt and Company; 2013.

[21] Haushofer J, Fehr E. On the psychology of poverty. Science. 2014;344(6186):862–7. 24855262 10.1126/science.1232491

[22] Fields SA, Lange K, Ramos A, Thamotharan S, Rassu F. The relationship between stress and delay discounting: a meta-analytic review. Behav Pharmacol. 2014;25(5–6):434–44. 25089842 10.1097/FBP.0000000000000044

[23] Schwabe L, Wolf OT. Stress prompts habit behavior in humans. J Neurosci. 2009;29(22):7191–8. 19494141 10.1523/JNEUROSCI.0979-09.2009 PMC6666491

[24] Vogel C, Ntani G, Inskip H, Barker M, Cummins S, Cooper C, et al. Education and the Relationship between Supermarket Environment and Diet. Am J Prev Med. 2016;51(2):E27–34. 27067035 10.1016/j.amepre.2016.02.030 PMC4959574

[25] Schultz S, Cameron AJ, Grigsby-Duffy L, Robinson E, Marshall J, Orellana L, Sacks G. Availability and placement of healthy and discretionary food in Australian supermarkets by chain and level of socio-economic disadvantage. Public Health Nutr. 2021;24(2):203–14. 32792022 10.1017/S1368980020002505 PMC10195512

[26] Schuz B, Meyerhof H, Hilz LK, Mata J. Equity effects of Dietary Nudging Field experiments: systematic review. Front Public Health. 2021;9:668998. 34368049 10.3389/fpubh.2021.668998 PMC8342848

[27] Langfield T, Marty L, Inns M, Jones A, Robinson E. Healthier diets for all? A systematic review and meta-analysis examining socioeconomic equity of the effect of increasing availability of healthier foods on food choice and energy intake. Obes Rev 2023;28;e13565([Online ahead of print]). 10.1111/obr.13565 36978200

[28] Vogel C, Piernas C. The Retail Food Environment. CRC; 2022. p. 16.

[29] Stuber JM, Lakerveld J, Kievitsbosch LW, Mackenbach JD, Beulens JW. Nudging customers towards healthier food and beverage purchases in a real-life online supermarket: a multi-arm randomized controlled trial. BMC Med. 2022;20(1):1–13. 35034635 10.1186/s12916-021-02205-z PMC8762859

[30] Neve KL, Coleman P, Hawkes C, Vogel C, Isaacs A. What shapes parental feeding decisions over the first 18 months of parenting: insights into drivers towards commercial and home-prepared foods among different socioeconomic groups in the UK. Appetite. 2024;196. 10.1016/j.appet.2024.107260 38403201

[31] Ayala GX, Pickrel JL, Baquero B, Sanchez-Flack J, Lin SF, Belch G et al. The El Valor De Nuestra Salud clustered randomized controlled trial store-based intervention to promote fruit and vegetable purchasing and consumption. Int J Behav Nutr Phy. 2022;19(1). 10.1186/s12966-021-01220-w PMC8851758 35177070

[32] Stuber JM, Mackenbach JD, de Bruijn GJ, Gillebaart M, Hoenink JC, Middel CNH, et al. Real-world nudging, pricing, and mobile physical activity coaching was insufficient to improve lifestyle behaviours and cardiometabolic health: the Supreme Nudge parallel cluster-randomised controlled supermarket trial. BMC Med. 2024;22(1):52. 38303069 10.1186/s12916-024-03268-4 PMC10835818

[33] Hoenink JC, Mackenbach JD, Waterlander W, Lakerveld J, Van Der Laan N, Beulens JW. The effects of nudging and pricing on healthy food purchasing behavior in a virtual supermarket setting: a randomized experiment. Int J Behav Nutr Phy. 2020;17(1):1–12. 10.1186/s12966-020-01005-7 PMC7398383 32746928

[34] Ejlerskov K, Sharp SJ, Stead M, Adamson AJ, White M, Adams J. Socio-economic and age variations in response to supermarket-led checkout food policies: a repeated measures analysis. Int J Behav Nutr Phys Act. 2018;15(1):125. 30518393 10.1186/s12966-018-0755-4 PMC6282373

[35] Boyle PA, Yu L, Wilson RS, Gamble K, Buchman AS, Bennett DA. Poor Decision Making Is a Consequence of Cognitive Decline among Older Persons without Alzheimer’s Disease or Mild Cognitive Impairment. PLoS ONE. 2012;7(8). 10.1371/journal.pone.0043647 PMC3423371 22916287

[36] Vogel C, Dijkstra C, Huitink M, Dhuria P, Poelman MP, Mackenbach JD, et al. Real-life experiments in supermarkets to encourage healthy dietary-related behaviours: opportunities, challenges and lessons learned. Int J Behav Nutr Phys Act. 2023;20(1):73. 37340326 10.1186/s12966-023-01448-8 PMC10280909

[37] Bangdiwala SI, Bhargava A, O’Connor DP, Robinson TN, Michie S, Murray DM, et al. Statistical methodologies to pool across multiple intervention studies. Transl Behav Med. 2016;6(2):228–35. 27356993 10.1007/s13142-016-0386-8 PMC4927450

[38] Fortier I, Raina P, Van den Heuvel ER, Griffith LE, Craig C, Saliba M, et al. Maelstrom Research guidelines for rigorous retrospective data harmonization. Int J Epidemiol. 2017;46(1):103–5. 27272186 10.1093/ije/dyw075 PMC5407152

[39] Stuber JM, Beulens JJW, Ayala GX, Crozier S, Dijkstra C, Lin SF et al. Pre-registration: Do sociodemographic characteristics moderate the effectiveness of nudge interventions on healthy food purchases in real-world grocery stores? An individual participant data meta-regression of four controlled trials: Open Science Framework registries 2023 [ 10.17605/OSF.IO/QJNKC

[40] NAICS Association. NAICS Code Description: 445110 - Supermarkets and Other Grocery (except Convenience) Stores [ https://www.naics.com/naics-code-description/?code=445110

[41] Vogel C, Crozier S, Penn-Newman D, Ball K, Moon G, Lord J, et al. Altering product placement to create a healthier layout in supermarkets: outcomes on store sales, customer purchasing, and diet in a prospective matched controlled cluster study. PLoS Med. 2021;18(9):e1003729. 34491999 10.1371/journal.pmed.1003729 PMC8423266

[42] Huitink M, Poelman MP, van den Eynde E, Seidell JC, Dijkstra SC. Social norm nudges in shopping trolleys to promote vegetable purchases: a quasi-experimental study in a supermarket in a deprived urban area in the Netherlands. Appetite. 2020:104655. 10.1016/j.appet.2020.104655 32247896

[43] Stuber JM, Mackenbach JD, de Boer FE, de Bruijn GJ, Gillebaart M, Harbers MC, et al. Reducing cardiometabolic risk in adults with a low socioeconomic position: protocol of the Supreme Nudge parallel cluster-randomised controlled supermarket trial. Nutr J. 2020;19(1):46. 32429917 10.1186/s12937-020-00562-8 PMC7236937

[44] Hollands GJ, Bignardi G, Johnston M, Kelly MP, Ogilvie D, Petticrew M et al. The TIPPME intervention typology for changing environments to change behaviour. Nat Hum Behav 2017;8(1).

[45] Fisher DJ, Copas AJ, Tierney JF, Parmar MK. A critical review of methods for the assessment of patient-level interactions in individual participant data meta-analysis of randomized trials, and guidance for practitioners. J Clin Epidemiol. 2011;64(9):949–67. 21411280 10.1016/j.jclinepi.2010.11.016

[46] Egger M, Smith GD, Phillips AN. Meta-analysis: principles and procedures. BMJ. 1997;315(7121):1533–7. 9432252 10.1136/bmj.315.7121.1533 PMC2127925

[47] Bender R, Lange S. Adjusting for multiple testing–when and how? J Clin Epidemiol. 2001;54(4):343–9. 11297884 10.1016/s0895-4356(00)00314-0

[48] Stuber JM, Hoenink JC, Beulens JWJ, Mackenbach JD, Lakerveld J. Shifting toward a healthier dietary pattern through nudging and pricing strategies: a secondary analysis of a randomized virtual supermarket experiment. Am J Clin Nutr. 2021;0(0):1–10. 10.1093/ajcn/nqab057 PMC8326041 33829225

[49] Vogel C, Abbott G, Ntani G, Barker M, Cooper C, Moon G et al. Examination of how food environment and psychological factors interact in their relationship with dietary behaviours: test of a cross-sectional model. Int J Behav Nutr Phy. 2019;16. 10.1186/s12966-019-0772-y PMC6354411 30700323

[50] Black C, Moon G, Baird J. Dietary inequalities: what is the evidence for the effect of the neighbourhood food environment? Health Place. 2014;27:229–42. 24200470 10.1016/j.healthplace.2013.09.015 PMC4948665

[51] WHO. European Regional obesity Report 2022. Copenhagen: WHO Regional Office for Europe; 2022.

[52] Jacobson MF, Krieger J, Brownell KD. Potential policy approaches to address Diet-Related diseases. JAMA. 2018;320(4):341–2. 29955819 10.1001/jama.2018.7434

[53] A Focus on Healthy Food in Retail Environments Baltimore. Johns Hopkins Bloomberg School of Public Health 2020 [ https://americanhealth.jhu.edu/news/focus-healthy-food-retail-environments

[54] Muir S, Dhuria P, Roe E, Lawrence W, Baird J, Vogel C. UK government’s new placement legislation is a ‘good first step’: a rapid qualitative analysis of consumer, business, enforcement and health stakeholder perspectives. BMC Med. 2023;21(1). 10.1186/s12916-023-02726-9 PMC9878939 36703194

[55] Moore S, Butler T. UK government delays restriction of promotions on less-healthy foods: serious implications for tackling obesity. Obesity. 2022;30(9):1722–3. 35899320 10.1002/oby.23524 PMC9546114

[56] Sanchez-Flack J, Baquero B, Lin SF, Belch G, Pickrel JL, Anderson CAM et al. Evaluation of Store Environment changes of an In-Store intervention to promote fruits and vegetables in Latino/Hispanic-Focused food stores. Int J Env Res Pub He. 2020;17(1). 10.3390/ijerph17010065 PMC6981808 31861788

