# Supplementary material for: Can nudge interventions targeting healthy food purchases in real-world grocery stores reduce diet-related health disparities? A pooled analysis of four controlled trials
Source: Int J Behav Nutr Phys Act. 2024 Dec 3;21:137. doi: 10.1186/s12966-024-01687-3 (PMC11616344; doi:10.1186/s12966-024-01687-3)
Supplement: Supplementary file 1 — Additional file 1: Supplementary Text 1. Search string used for literature search in PubMed; Table S1. Recoding scheme to generate harmonised dataset; Table S2. Data re-structuring into a longitudinal and a cross-sectional data frame; Table S3. Study population characteristics in the cross-sectional analysis1 (n = 855); Figure S1. Flow diagram of exclusions in the longitudinal analysis for the fruit and vegetable purchases outcome; Figure S2. Flow diagram of exclusions in the longitudinal analysis for the diet quality outcome; Figure S3. Flow diagram of exclusions in the cross-sectional data frame for the fruit and vegetable purchases outcome; Figure S4. Trial-specific and overall pooled within- and between group nudge effectiveness on the percentage of fruit and vegetables purchased, based on the longitudinal data frame; Figure S5. Trial-specific and overall pooled between group nudge effectiveness on the percentage of fruit and vegetables purchased, based on the cross-sectional data frame; Figure S6. Trial-specific and overall pooled within- and between group nudge effectiveness on diet quality, based on the longitudinal data frame [file 12966_2024_1687_MOESM1_ESM.docx]

**Can nudge interventions targeting healthy food purchases in real-world grocery stores reduce socioeconomic health inequalities? A pooled analysis of four controlled trials**

J. M. Stuber

*Supplementary materials*

[Supplementary text 2](#_Toc168995078)

[1. Search string used for literature search in PubMed 2](#_Toc168995079)

[Supplementary tables 3](#_Toc168995080)

[Table S1. Recoding scheme to generate harmonised dataset 3](#_Toc168995081)

[Table S2. Data re-structuring into a longitudinal and a cross-sectional data frame 5](#_Toc168995082)

[Table S3. Study population characteristics in the cross-sectional analysis^1^ (n=855) 6](#_Toc168995083)

[Supplementary figures 7](#_Toc168995084)

[Figure S1. Flow diagram of exclusions in the longitudinal analysis for the fruit and vegetable purchases outcome 7](#_Toc168995085)

[Figure S2. Flow diagram of exclusions in the longitudinal analysis for the diet quality outcome 8](#_Toc168995086)

[Figure S3. Flow diagram of exclusions in the cross-sectional data frame for the fruit and vegetable purchases outcome 9](#_Toc168995087)

[Figure S4. Trial-specific and overall pooled within- and between group nudge effectiveness in the intervention group compared to the control group on the percentage of fruit and vegetables purchased, based on the longitudinal data frame 10](#_Toc168995088)

[Figure S5. Trial-specific and overall pooled between group nudge effectiveness in the intervention group compared to the control group on the percentage of fruit and vegetables purchased, based on the cross-sectional data frame 11](#_Toc168995089)

[Figure S6. Trial-specific and overall pooled within- and between group nudge effectiveness in the intervention group compared to the control group on diet quality (z-score), based on the longitudinal data frame 12](#_Toc168995090)

# Supplementary text

## 1. Search string used for literature search in PubMed

(supermarket*[ti] OR store*[ti] OR retail*[ti] OR grocery*[ti])
 AND
(nudge*[tiab] OR "choice architecture"[tiab] OR “social marketing”[tiab] OR prompt*[tiab] OR swap*[tiab] OR position*[tiab] OR placement*[tiab] OR promotion*[tiab] OR label[tiab] OR shelf[tiab])
 AND
(intake*[tiab] OR diet*[tiab] OR sales*[tiab] OR purchase*[tiab] OR groceries[tiab] OR food[tiab] OR beverages[tiab])
 AND
(controlled[tiab] OR matched[tiab] OR "quasi experimental"[tiab])
 NOT
("natural experiment"[tiab] OR online[tiab] OR virtual*[tiab] OR simulated*[tiab] OR laboratory*[tiab])

This search resulted in 47 hits on March 30, 2023, and 53 hits after an updated search on April 19, 2024. No new relevant trials were identified via the second search.

# Supplementary tables

## Table S1. Recoding scheme to generate harmonised dataset

| **Harmonized variable names** | **Operationalisation harmonized variables** | **Ayala – original variables** | **Huitink – original variables** | **Vogel – original variables** | **Stuber – original variables** |
| --- | --- | --- | --- | --- | --- |
| ***Identification variables*** | | | | | |
| Trial | Nominal scale:  1 = Ayala  2 = Huitink  3 = Vogel  4 = Stuber | N/A | N/A | N/A | N/A |
| Participants | Recode with random values on nominal scale | Nominal values | Nominal values | Nominal values | Nominal values |
| Grocery_stores | Recode with random values on nominal scale | Nominal values for 16 grocery store locations | N/A | Nominal values for 6 grocery store locations | Nominal values for 12 grocery store locations |
| Group | Dichotomous scale:  1 = Control  2 = Intervention | Dichotomous:  0 = Control  1 = Intervention | Dichotomous:  0 = Intervention  1 = Control | Dichotomous:  1 = Control  2 = Intervention | Dichotomous:  0 = Control  1 = Intervention |
| ***Moderating variables*** | | | | | |
| Education | Dichotomous scale:  1 = Lower (low and medium)  2 = Higher (high) | Categorical scale, educational attainment:  1= 6 years or less (low)  2 = 7-11 years (medium)  3 = high school degree or more (high) | Categorical scale, education:  0 = Do not want to disclose  1 = Lager Onderwijs (primary/low)  2 = Lbo (primary/low)  3 = Mavo (secondary/medium)  4 = Mbo (secondary/medium)  5 = Havo/Vwo (secondary/medium)  6 = Hbo (tertiary/high)  7 = WO (tertiary/high) | Categorical scale, educational qualification:  1= Low (no qualifications beyond age 16)  2 = Medium  3 = High | Categorical scale, educational attainment:  1 = Low (no education and primary)  2 = Medium (secondary)  3 = High (tertiary) |
| Sex | Dichotomous scale:  1 = Male  2 = Female | Dichotomous scale:  0 = Female  1 = Male | Dichotomous scale:  0 = Male  1 = Female | Females-only sample | Dichotomous scale:  1 = Female  2 = Male |
| Age | Dichotomous scale:  1 = Younger adults (18 – 55 years)  2 = Older adults (>55 years) | Continuous scale in years | Dichotomous scale:  1 = 18-55 years  2 = >55 years | Continuous scale in years | Continuous scale in years |
| ***Outcome variables*** | | | | | |
| Percentage_FV _purchased | Continuous scale: 0-100, reflecting the percentage of fruits and vegetables purchased in dollars/items/grams of all dollars/items/grams spent. | Continuous scales:  - Total dollars spent;  - Dollars spent on fruit and vegetables;  - Percentage of dollars spent on fruit and vegetables from total dollars spent on groceries. | Continuous scales:  - Vegetables purchased in items;  - Total number of items purchased;  - Percentage of vegetable items purchased of all items purchased. | Continuous scales:  - Fruit and vegetables purchased in items;  - Total number of items purchased. | Continuous scales:  - Fruit and vegetables purchased in grams;  - Total number of grams purchased;  - Percentage of fruit and vegetable grams purchased of all grams purchased. |
| Diet_quality | Continuous scale: z-scores | N/A | N/A | Continuous scale: Overall diet quality (z-scores) | Continuous scale: Overall diet quality (Dutch Healthy Diet index, scored 0-150) |
| ***Confounding variables*** | |  |  |  |  |
|  | Confounding variables available across trials, which are used in the current main analyses:  Sex, age, education, number of persons purchased groceries for (for trials by Vogel et al. and Stuber et al.: ‘Household size’ was labelled as ‘Number of persons purchased for’). | - Non-Hispanic score^1^ (continuous);  - Poverty (dichotomous);  - Homeownership (dichotomous);  - Number of persons purchased for (count);  - Household size (count). | - Sex (dichotomous);  - Age (continuous);  - Education (categorical);  - Number of persons purchased groceries for (count). | - Age (continuous);  - Education (categorical);  - Money spent on groceries (continuous);  - Number of children in the household (count)  *Note: data on total household size is available.* | N/A  *Note: data on household size is available.* |

^1^Non-Hispanic score is a measure of acculturation among immigrants to the US from Spanish-speaking countries.

## Table S2. Data re-structuring into a longitudinal and a cross-sectional data frame

|  | **Operationalisation harmonised dataset** | **Ayala – original time points** | **Huitink – original time points** | **Vogel – original time points** | **Stuber – original time points** |
| --- | --- | --- | --- | --- | --- |
| **Original time points** | N/A | Measurements at baseline, after 6 months, and 12 months. | Outcome data collected at two intervention days and two control days over a two week period. | Measurements at baseline, after 3 months, and 6 months. | Measurements at baseline, after 3 months, 6 months, and 12 months (diet quality data) and averages per four weeks over a 12-month trial period (purchase data). |
| **Longitudinal data frame:**  Time in long frame,  for longitudinal within- and between group analysis | Categorical scale:  0 = Baseline  1 = 3 months  2 = 6 months  3 = 12 months | Categorical scale:  0 = Baseline  1 = Not collected  2 = 6 months  3 = 12 months | N/A; data is excluded from data frame 1. | Categorical scale:  0 = Baseline  1 = 3 months  2 = 6 months  3 = Not collected | Categorical scale:  0 = Baseline  1 = 3 months  2 = 6 months  3 = 12 months |
| **Cross-sectional data frame:**  Time in wide frame, for between group difference analysis | Time variable is omitted, instead data is analysed by the Group variable; dichotomous scale:  1 = Control  2 = Intervention | Only use data collected at month 6, dichotomous scale:  0 = Control  1 = Intervention | Dichotomous scale:  0 = Control  1 = Intervention | Only use data collected at month 6, dichotomous scale:  1 = Control  2 = Intervention | Only use data collected at month 6, dichotomous scale:  0 = Control  1 = Intervention |

## Table S3. Study population characteristics in the cross-sectional analysis^1^ (n=855)

|  | **Control group**  **(n=434)** | | **Intervention group**  **(n=421)** | |
| --- | --- | --- | --- | --- |
| Participants within trials, n (%) |  |  |  |  |
| *Trial by Ayala et al.* | 161 | (37.1) | 169 | (40.1) |
| *Trial by Huitink et al.* | 101 | (23.3) | 114 | (27.1) |
| *Trial by Vogel et al.* | 47 | (10.8) | 40 | (9.5) |
| *Trial by Stuber et al.* | 125 | (28.8) | 98 | (23.3) |
| Educational attainment^2^, n (%) |  |  |  |  |
| *Lower* | 257 | (59.2) | 258 | (61.3) |
| *Higher* | 177 | (40.8) | 163 | (38.7) |
| Sex, n (%) |  |  |  |  |
| *Females* | 307 | (70.7) | 315 | (74.8) |
| *Males* | 127 | (29.3) | 106 | (25.2) |
| Age, n (%) |  |  |  |  |
| *Younger adults (18 – 55 years)* | 266 | (61.3) | 283 | (67.2) |
| *Older adults (>55 years)* | 168 | (38.7) | 138 | (32.8) |
| Age^3^, mean (SD) | 48.2 | (13.8) | 46.2 | (13.1) |
| Number of persons purchased groceries for, median [IQR] | 3.0 | [2.0] | 3.0 | [2.0] |
| Percentage fruit and vegetable purchases of total purchases, median [IQR] | 22.2 | [22.1] | 20.0 | [22.5] |

^1^The cross-sectional analysis includes data from all four included trials, incorporating data collected at month 6 of each of the three longitudinal trials to compare intervention versus control data at a single time point; ^2^Lower = low and medium level educational attainment, higher =higher educational attainment; ^3^Based on n=640 due to absence of continuous data on age in the dataset by Huitink et al.

# Supplementary figures


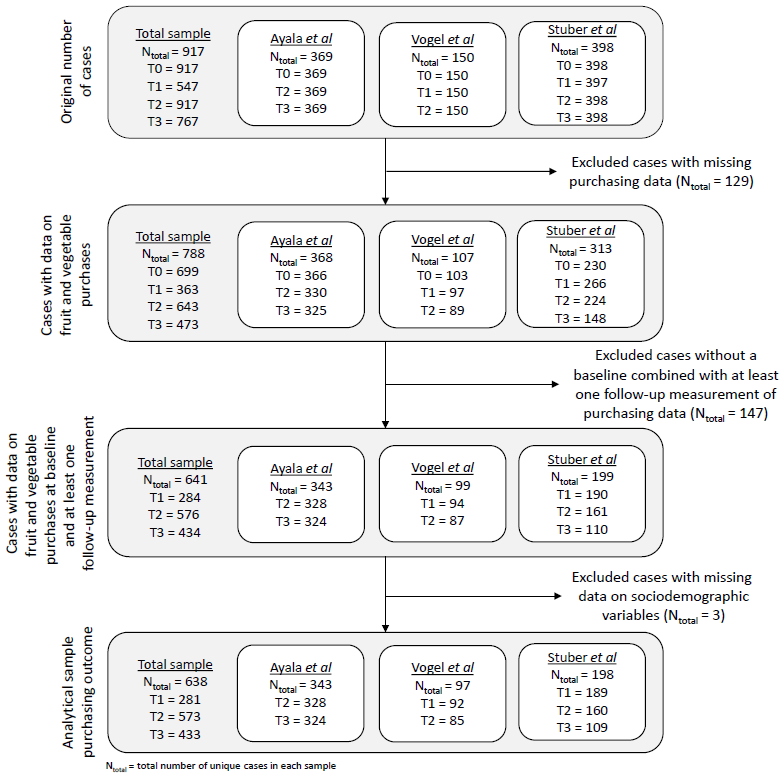


## Figure S1. Flow diagram of exclusions in the longitudinal analysis for the fruit and vegetable purchases outcome


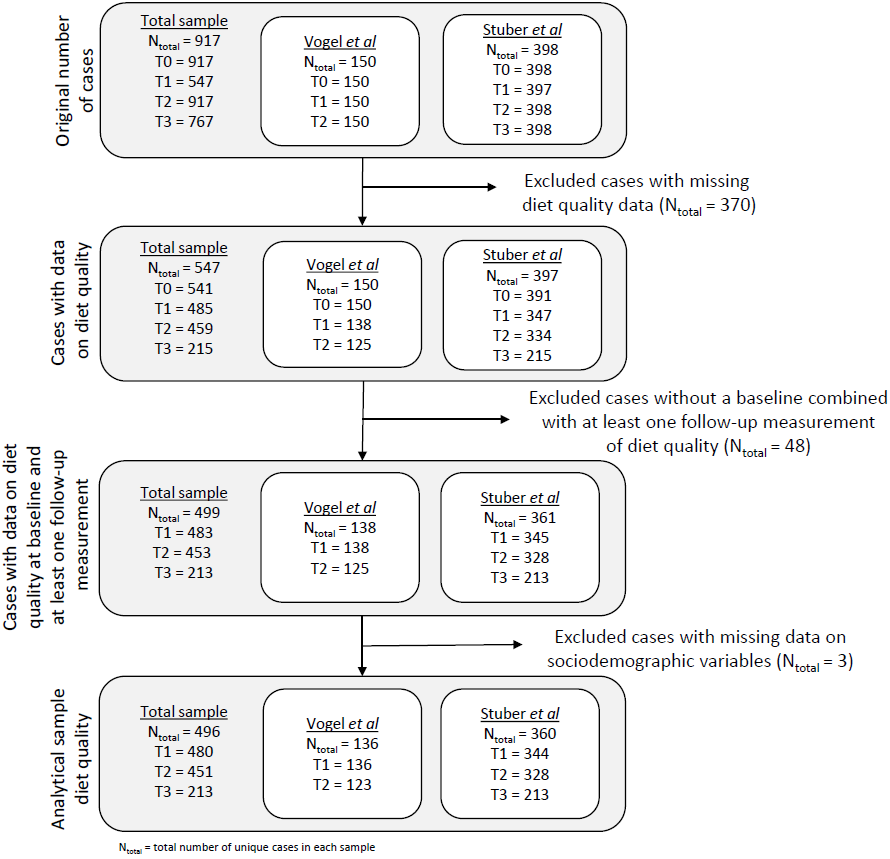


## Figure S2. Flow diagram of exclusions in the longitudinal analysis for the diet quality outcome


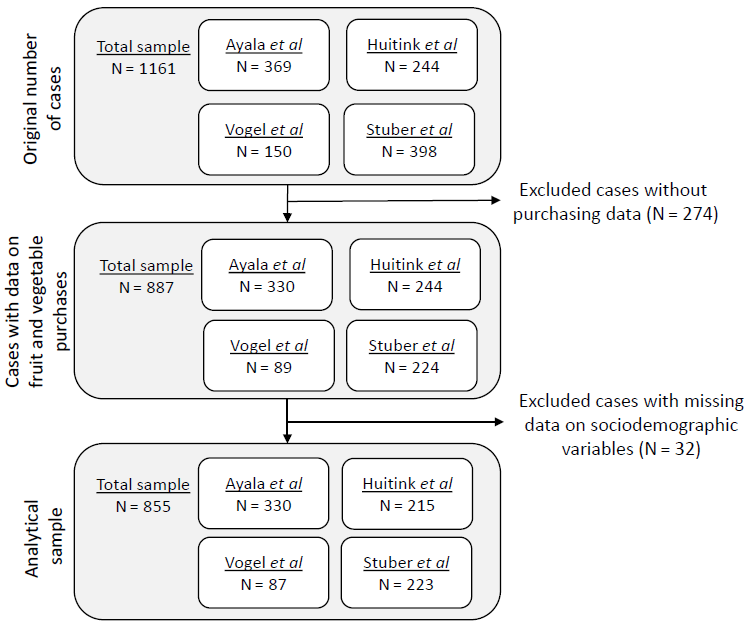


## Figure S3. Flow diagram of exclusions in the cross-sectional data frame for the fruit and vegetable purchases outcome


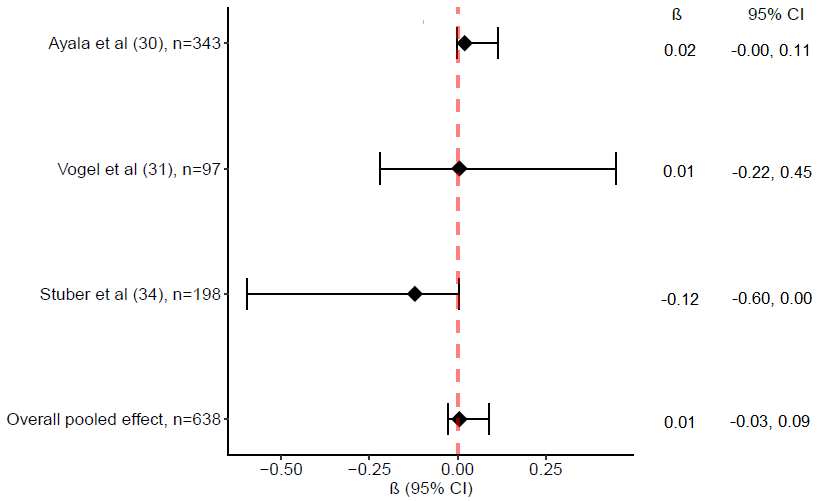


## Figure S4. Trial-specific and overall pooled within- and between group nudge effectiveness in the intervention group compared to the control group on the percentage of fruit and vegetables purchased, based on the longitudinal data frame

Analyses were based on linear mixed-effects models, with group as independent variable and the square root of the percentage of fruit and vegetable purchases as the dependent variable. Further fixed effects were time (categorical) and the baseline measurement of percentage of fruit and vegetable purchases. Random intercepts were included for trials (only in the pooled effects model), grocery stores, and participants. Models were adjusted for educational attainment, sex, age, and the number of persons purchased groceries for. Results reflect back-transformed means from the square root transformations.


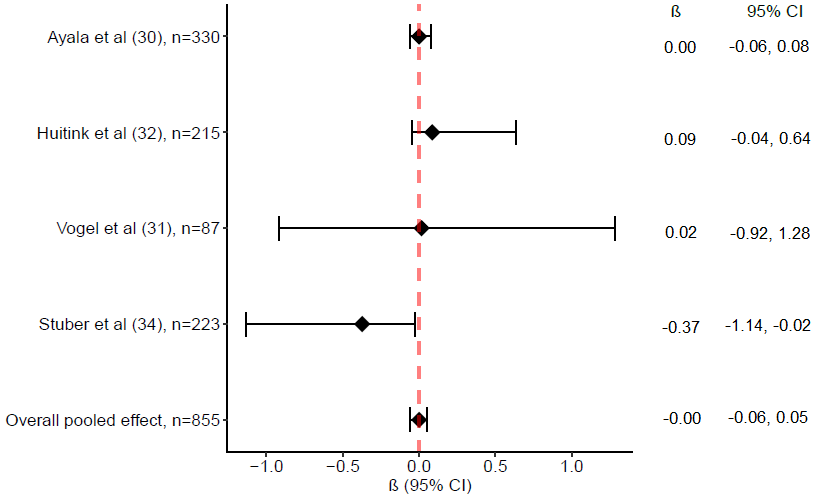


## Figure S5. Trial-specific and overall pooled between group nudge effectiveness in the intervention group compared to the control group on the percentage of fruit and vegetables purchased, based on the cross-sectional data frame

Analyses were based on linear mixed-effects models, with group as independent variable and the square root of the percentage of fruit and vegetable purchases as the dependent variable. Random intercepts were included for trials (only in the pooled effects model), and grocery stores. An exception was the trial by Huitink et al, which consisted of data from a single store. The analyses of these data was thus based on a linear model without a random intercept for stores. All models were adjusted for educational attainment, sex, age, and the number of persons purchased groceries for. Results reflect back-transformed means from the square root transformations.


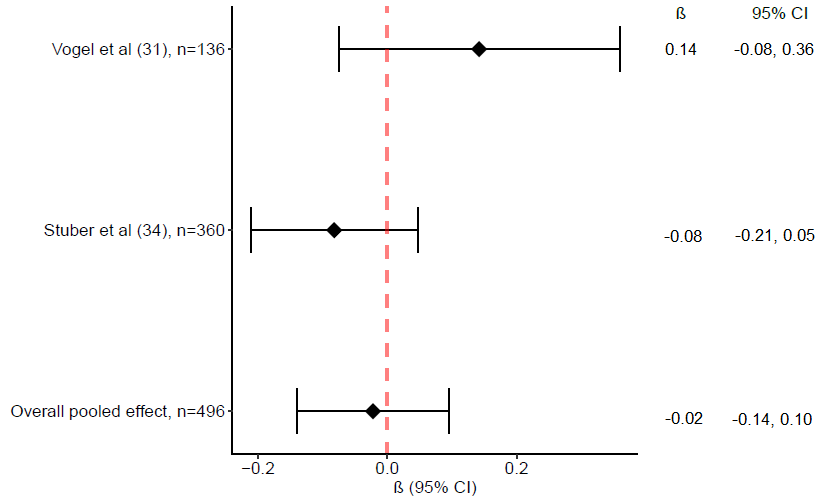


## Figure S6. Trial-specific and overall pooled within- and between group nudge effectiveness in the intervention group compared to the control group on diet quality (z-score), based on the longitudinal data frame

Analyses were based on linear mixed-effects models, with group as independent variable and diet quality as the dependent variable. Further fixed effects were time (categorical) and the baseline measurement of diet quality. Random intercepts were included for trials (only in the pooled effects model), grocery stores, and participants. Models were adjusted for educational attainment, sex, age, and the number of persons purchased groceries for.
